# Intensive aerobic and muscle endurance exercise in patients with systemic sclerosis: a pilot study

**DOI:** 10.1186/1756-0500-7-86

**Published:** 2014-02-07

**Authors:** Helene Alexanderson, Jenny Bergegård, Lena Björnådal, Annica Nordin

**Affiliations:** 1 Department of Neurobiology, Care Science and Society, Division of Physical Therapy, Karolinska Institutet, Stockholm, Sweden; 2 Department of Physical Therapy, Orthopedic/Rheumatology Unit, Karolinska University Hospital, Stockholm, Sweden; 3 Department of Medicine, Rheumatology Unit, Karolinska Institutet, Stockholm, Sweden

**Keywords:** Systemic sclerosis, Physical exercise, Rehabilitation, Aerobic capacity, Interstitial lung disease

## Abstract

**Background:**

No previous studies have examined the effect of intensive exercise in systemic sclerosis patients with pulmonary impairment. The objective of this study was to examine the effect of an eight-week intensive aerobic exercise and muscle endurance training program for patients with systemic sclerosis with 50–100% of forced vital capacity.

**Methods:**

A single-subject experimental design with repeated systematic measures during a six week A-phase (non-interventional baseline period) and an eight week B-phase (exercise intervention period) was used. Three women and one man with median age 66 years and median disease duration of 3.5 years completed aerobic exercise corresponding to 15 on the Borg RPE scale (strenuous) and muscular endurance training three times/week. Physical capacity (six-minute walk test), aerobic capacity (submaximal treadmill test) and muscle endurance in shoulder and hip flexion (Functional Index 2) were assessed every other week throughout the 14-week study. Activity limitation (Health Assessment Questionnaire), quality of life (Short Form 36), Raynaud, Fatigue and Global Health during the recent week (Visual Analogue Scales) were assessed at weeks 0, 6, 14.

**Results:**

Three participants improved significantly in muscular endurance, and two participants improved significantly or clinically relevant in aerobic capacity. All other variables remained unchanged, except for a trend towards reduced fatigue.

**Conclusions:**

This eight week exercise program was largely successful with positive effects on aerobic capacity and muscle endurance.

**Trial registration:**

Clinicaltrials.gov Identifier: NCT01813578

## Background

Systemic sclerosis (SSc) is an uncommon autoimmune systemic disorder presenting with skin fibrosis and vasculopathy and involving several different organ systems. Based on the degree of skin involvement, the disease is subdivided into limited cutaneous SSc (lcSSc) or diffuse cutaneous SSc (dcSSc). Aside from skin fibrosis, Raynaud’s phenomena and gastrointestinal involvement are the most common disease manifestations [1].

Interstitial lung disease (ILD) is common, especially in dcSSc, and along with pulmonary hypertension is the main cause of death in these patients [2]. Cardiopulmonal involvement, when present, leads to shortness of breath and reduced aerobic capacity [3]. In addition, arthralgia and arthritis are common problems and patients often experience reduced muscle endurance and fatigability [4]. Individuals have self-reported reduced physical function [5,6] and poorer quality of life compared to the general population [7-9].

So far, only four studies have evaluated different exercise regimens in SSc. All indicate the safety of exercise with reduced disability. One study reported significantly improved aerobic capacity in a group of seven patients with SSc without lung involvement and seven healthy controls following eight weeks of intensive aerobic exercise [10]. An open study of 12 patients with SSc without ILD reported improved muscle strength following an intensive 12-week aerobic and muscle training program in 12 patients with SSc without ILD [11]. A randomized controlled trial involving 20 patients with SSc evaluated the effects of a rehabilitation program with manual techniques such as massage and joint mobilization in combination with aquatic training, including walking, stretching and breathing exercises performed twice a week for nine weeks [12]. Patients with some degree of lung involvement were included in both groups. The treatment group improved significantly in self-reported quality of life and activity limitation [12]. An individualized rehabilitation program was evaluated involving 16 patients of whom seven had lung fibrosis [13]. The group performed breathing exercises, aerobic exercise on a treadmill or outdoor walks, and 10 individual rehabilitation sessions during a two-week period. Patients were then instructed to perform a home exercise program during the four-month follow-up period. After four months, the exercise group had improved in self-reported quality of life and hand functions, and had reduced exercise heart rates and lowered perceived exertions during the six-minute walking test while the non-randomized control group remained unchanged [13].

Today evidence for the safety and efficacy of aerobic and resistance training in patients with other inflammatory diseases supports the inclusion of exercise as an important part of the treatment of these patients [14,15]. Further, aerobic exercise has been reported to improve walking distance and quality of life as well as to reduce dyspnea in patients with ILD [16]. So far evidence for safety and effects of exercise in patients with SSc is limited, especially regarding intensive training.

The aim of this study was to evaluate the effects of eight weeks of intensive aerobic exercise and muscle endurance training regarding walking ability, muscle function, aerobic capacity, limitations in daily activities and quality of life in patients with limited or diffuse SSc with 50–100% of vital capacity.

## Methods

Patients were recruited to this present study from an ongoing screening study for cardiovascular disease in SSc at the Rheumatology clinic at Karolinska University Hospital, Stockholm, Sweden. Medical charts were reviewed and all patients fulfilling the criteria were invited to participate in this exercise study (n = 15). The inclusion criteria were; limited or diffuse SSc, age 18–80 years, diagnosis duration ≥ 1 year, unchanged medication for 3 months, 50–100% of normal vital capacity, exercising ≤ once a week, Swedish language fluency, average or below aerobic capacity according to the submaximal treadmill test (≤ 27 ml/kg × min for women and ≤ 30 ml/kg × min for men) [17], ≤ 20% reduced muscle endurance according to the Functional Index-2. The exclusion criteria; Cyclophosphamide treatment for alveolitis, Pulmonal arterial hypertension (PAH), heart condition contraindicating exercise, < 50% of normal vital capacity, reduced kidney function. Eleven patients declined participation, due to lack of time in 10 cases, and a long trip abroad in one case.

Thus, four patients participated, three women and one man with a median age of 66.5 years, ranging from (41–69) years old with a median 3.5 range (2–5) years diagnosis duration. Demographic data are presented in Table 1. Two participants had lung fibrosis, participant 1 and 2, with 50 and 80% of forced vital capacity (FVC), respectively, while the other two had 100% FVC. Two were treated with low dose of oral corticosteroids, one with mycophenolate mofetil, and another one with statins. One was further treated with estrogen and bronchial dilation. None of the participants exercised during the six months preceding inclusion in the study, but three patients took ordinary walks 3–14 times per week. One was not physically active at all. They had all retired from working life.

**Table 1:** **Fatigue**, **Raynaud**’**s phenomenon**, **patients**’ **global assessment of disease and perceived health assessed by the SF**-**36 in the 4 participants**

**Participants**	**1**	**2**	**3**	**4**
**Assessment time points**	**0w**	**0w**	**0w**	**0w**
**Fatigue, VAS, 0-100**	72	50	83	4
**Raynaud, VAS, 0-100**	56	55	60	50
**Patient’s Global, VAS, 0-100**	46	51	33	35
**HAQ, 0**-**3**	0.50	0.13	0.50	0.50
**SF-36, 0-100**				
Physical functioning	60	45	30	60
Role physical	0	0	100	100
Bodily pain	22	100	41	62
General health	50	35	15	67
Vitality	25	60	15	85
Social functioning	25	100	75	75
Role emotional	33	33	100	100
Mental health	64	72	60	92

0w = first assessment time point of the A-phase.

6w = last assessment time point of the A-phase, right before starting exercise.

14w = last assessment point of the B-phase, after completion of the 8 weeks of exercise.

VAS = visual analogue scale.

HAQ = Stanford Health Assessment Questionnaire.

SF-36 = Short Form 36.

Demographic data such as age, gender, time of diagnosis, other diseases, medication, work, and ongoing physical therapy/exercise, as well as level of physical activity were registered in a questionnaire before the study started.

The six minute walk test (6MWT) was the primary outcome [18,19]. The participants were instructed to walk as far as possible at their pace of choice for six minutes and to stop and rest when needed. Heart rate, saturation, and perceived central and peripheral exertion according to the Borg CR-10 scale (0–10) [20] were recorded before starting the test at rest and after 2, 4, and 6 minutes of walking. The participants were asked to rate general exertion according to the Borg RPE-scale (6–20) [21] at the completion of the test.

A submaximal treadmill test was used to estimate oxygen uptake in ml/kg × min [17]. The test requires a total of eight minutes where the first four minutes are performed on a horizontal treadmill with the participant walking at a self-selected walking speed. During the following four minutes, the patient continues at the same walking speed on a 5% incline. Working heart rate, saturation, and central and peripheral exertion according to the Borg RPE 6–20 scale [21] were recorded every other minutes and after completion of the test.

The number of correctly performed repetitions of the shoulder and hip flexion tasks included in the Functional Index 2 [22] was recorded as a measure of muscle endurance. Maximal numbers of repetitions are 60 for both tasks, representing no limitations. After completing each task, the perceived muscle exertion was recorded according to the Borg CR-10 scale [20].

A Visual Analogue Scale (VAS) of 0–100 was used to assess patients’ perceptions of Raynaud’s phenomenon, global fatigue and global disease impact on well-being during the last week [23].

The Stanford Health Assessment Questionnaire – HAQ was used to assess activity limitation [24]. The HAQ comprises 20 items divided into eight categories; dressing and grooming, arising, eating, walking, hygiene, reach, grip and other activity. The HAQ score varies from 0–3, where 0 indicates no limitation.

The SF-36 was used to assess perceived health comprising 36 items divided into eight domains; physical functioning, role physical, bodily pain, general health, vitality, social functioning, role emotional, and mental health [25]. Each domain is scored separately between 0–100, where 100 indicates good health.

As SSc is a rare condition, a Single Subject Experimental Design (SSED) was used where each participant is his or hers own control. According to an AB SSED, measures producing quote data (six-minute walking test, the treadmill test for estimated oxygen uptake and the muscle endurance test FI-2) were systematically performed during the four-week non-interventional A-phase and the following eight-week interventional B-phase [26]. Measures producing ordinal data (VAS), the HAQ and the SF-36 were performed at 0, 6 and 14 weeks.

All clinical assessments were performed by one trained physical therapist who was not involved in the exercise intervention.

The exercise program contained aerobic exercise on a stationary bike and muscular endurance training of the shoulder flexors and the hip flexors. The program started with a 10-minute warm-up biking session at an intensity corresponding to perceived light exertion (10 on the Borg RPE, 6–20 scale) [21]. Afterward, the load was increased to reach an intensity corresponding to perceived heavy exertion (15 on the Borg RPE scale) for an additional 15 minutes. During the last five minutes of the biking session, the loads were reduced to an intensity corresponding to light exertion. Heart rate was recorded with a pulse watch (RS100 Polar) and peripheral saturation was recorded with a heart rate oxymeter (Wristox 3100 Nonin Medical) during the biking exercise part of the program. During the first week, patients performed only the 10-minute warm-up exercise and the following week another 5 minutes were added with an intensity corresponding to somewhat exerting (13 on the Borg RPE scale). During the remaining six weeks, patients exercised with a goal intensity corresponding to perceived heavy exertion (15 on the Borg RPE scale) for a maximum of 30 minutes. After the biking exercises, the participants performed repetitive dynamic muscle resistance training with the goal of achieving as many shoulder flexion repetitions as possible in a sitting position without back support with a 1-kg weight cuff around the wrist, and in hip flexion, lifting one leg at a time while lying in a supine position. The numbers of repetitions were based on the results of the FI-2 tests for these muscle groups. If participants at some point during the exercise period performed the maximal number of repetitions (60) of shoulder flexion, a heavier cuff was attached to the wrist to increase the load. In case of performing maximal repetitions of hip flexion, a weight cuff of 0.5 – 1.0 kg was attached to the ankle. Every other week, the numbers of repetitions were adapted according to the results of the every other week systematic assessments. During the first two weeks, patients performed only 50% of the maximal number of repetitions performed at the 0-week assessment and then gradually increased to perform the maximal number of repetitions at each exercise session. The participants exercised three times a week under the supervision of a trained physical therapist at the Department of Physical Therapy. To enhance compliance, participants were given the choice to exercise at home or at a gym twice a week and once a week under supervision at the hospital.

According to the 2-standard deviation (2 SD) band analysis, statistical analyses were performed for each patient individually [27]. Accordingly, a statistically significant change was defined as two consecutive assessments in the B-phase exceeding the two standard deviations (SD) of the mean of the A-phase. To improve the internal and external validity, analysis from more than one participant needs to be performed [26,27]. Measures producing quote data are presented individually as mean and 2 SD for the A-phase and as actual values from each assessment time point in the B-phase. Measures producing ordinal data are presented individually and were analyzed using graphically identified trends or slope [26]. Where a trend was identified, the Friedman’s ANOVA was used. The statistical significance level was set to p < 0.05. The Statistica 10.0 was used. Patients were also analyzed according to responder criteria. Participants improving ≥ 20% in walking distance, aerobic capacity and/or muscle endurance compared to the A-phase mean value were considered as a responder [28]. This study was approved by the Karolinska University Hospital Research Ethics Committee and all participants signed informed consent forms before entering the study.

## Results

Three of the four participants (2, 3 and 4) completed all 24 exercise sessions as planned while participant 1 completed 22 sessions, missing two due to medical investigations of increased lung symptoms.

Participant 1 fulfilled all the inclusion criteria before entering the study, however the participant was unable to increase exercise intensity three weeks into the B-phase due to increased dyspnea and cough during exercise. New lung screening revealed increased bronchial obstructivity and increased lung fluid. After initiating diuretic and bronchial dilating treatment, the patient was cleared to continue the exercise program, though remaining on the intensity of the somewhat exerting exercise initiated at week three (13 on the Borg RPE). Two months after completing the exercise program, another lung screening revealed increased pulmonary disease activity along with increased skin symptoms. The diagnosis was changed from limited to diffuse SSc. Participant 2 was not able to complete the treadmill test during the A-phase assessments due to demonstrating maximal dyspnea, but was then able to complete the test during all B-phase assessments. Participant 3 had reduced postural control and reduced distal tactility of the lower limbs due to lumbar nerve impingement. Participant 3 was not able to perform the aerobic exercise due to intense radiating pain in the lower limbs after just one or two minutes of biking, but performed all aerobic exercise sessions on a treadmill, setting the exercise intensity according to the same perceived exertion protocol as the other participants. Participant 4 had fluctuating ankle arthritis pain which had been treated with corticosteroid injections four months before entering the study with sub-optimal results. This participant reached the maximal number of repetitions of shoulder flexion already during the A-phase and thus had no potential for improvement in this test. However, the patient was able to perform the 60 repetitions with 2 kg weights at the end of the study.

**Figure 1:**
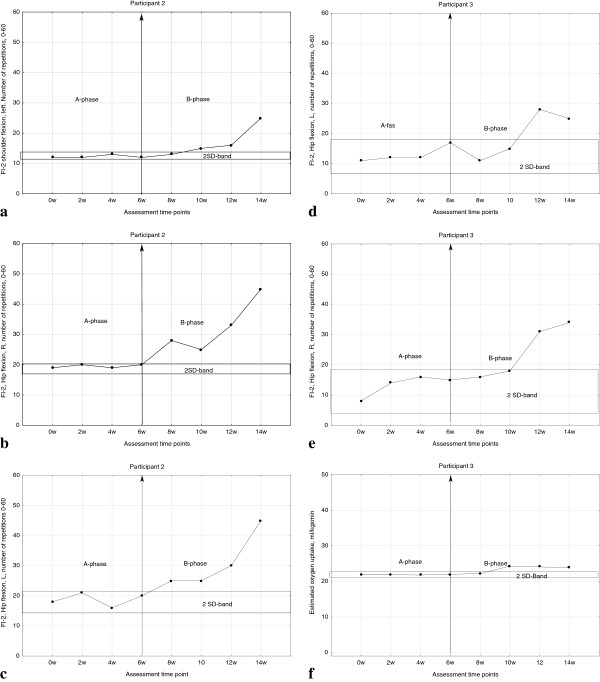
Individual results of muscle endurance assessed by the FI-2 in participants 2 and 3 (1a-1e), and aerobic capacity in participant 3 (1f).

Individual results of all variables are presented in Table 2. After eight weeks of exercise, no participant showed a statistically significant change in physical walking distance during the 6MWT. Participant 2 improved significantly in shoulder flexion muscle endurance (Figure 1a) while participants 2 and 3 improved significantly in hip flexion muscle endurance (Figure 1b-e). Participants 2 and 3 improved ≥20% in shoulder flexion muscle endurance bilaterally and participants 2, 3 and 4 improved in hip flexion bilaterally, according to criteria [29]. Participant 1 worsened significantly, but not ≥ 20%, in shoulder flexion, left side (Table 2). Participant 3 improved significantly in aerobic capacity assessed by the treadmill test after the eight-week exercise program (Figure 1f, Table 2). Participant 2 was not able to complete the test at any of the four A-phase assessment time points, enabling calculations of mean value and 2SD for the A-phase, but could complete all treadmill tests during the B-phase (Table 2).

**Table 2:** **A**-**phase mean and 2SD**, (**mean** + **2SD**), **and B**-**phase assessment points presented individually for each participant** (**1**,**2**,**3**,**4**)

	**1**				**2**				**3**				**4**			
	**A mean** **±** **2SD (mean** **+** **2SD)**				**A mean** **±** **2SD (mean** **+** **2SD)**				**A mean** **±** **2SD (mean** **+** **2SD)**				**A mean** **±** **2SD (mean** **+** **2SD)**			
	**B weeks**				**B weeks**				**B weeks**				**B weeks**			
	**8**	**10**	**12**	**14**	**8**	**10**	**12**	**14**	**8**	**10**	**12**	**14**	**8**	**10**	**12**	**14**
**6MWT, m**	527 ± 64 (591)				429 ± 59 (488)				384 ± 73 (457)				406 ± 23 (429)			
	510	543	484	537	455	392	423	450	390	390	394	407	421	424	390	420
**Treadmill,**	31.7 ± 5.8 (37.5)				α				21.9 ± 0.7 (22.6)				31.4 ± 1.5 (32.9)			
ml/kg x min	30.0	27.0	30.0	29.0	23.0	21.4	23.0	24.3#	22.2	24.3	24.1	23.8*	30.9	31.0	31.5	31.2
**FI-2, SF, R**	10 ± 5.4 (15.4)				13.3 ± 4 (17.3)				22.3 ± 4.8 (27.1)				60 ± 0 (60)			
Repetitions	9	7	10	10	16	16	17	29#	24	21	25	28#	60	60	60	60
**FI-2, SF, L**	9.5 ± 2 (11.5, 7.5) ¤				12.3 ± 1 (13.3)				19.5 ± 6.2 (25.7)				60 ± 0 (60)			
Repetitions	5	5	7	7	13	15	16	25*#	25	23	30	25#	60	60	60	60
**FI-2 HF, R**	7.8 ± 4.1 (11.9)				19.5 ± 1.2 (21.7)				13.3 ± 7.2 (20.5)				37.3 ± 25.4 (62.7)			
Repetitions	6	5	7	7	28	25	33	45*#	16	18	31	34*#	50	60	60	60#
**FI-2, HF, L**	8.5 ± 3.9 (12.4)				18.9 ± 4.4 (23.3)				13.0 ± 5.4 (18.4)				35.5 ± 21.3 (56.8)			
Repetitions	7	6	3	8	25	25	30	45*#	11	15	28	25*#	48	60	60	60*#

A = non-interventional A-phase, 2SD = 2 standard deviations from the mean, B = interventional B-phase, 6MWT = 6-minute walking test, FI-2 = Functional Index-2, SF = Shoulder flexion, HF = Hip flexion, R = Right side, L = Left side, α = participant could not complete any of the A-phase assessments due to exertion, * = at least 2 consecutive assessments in the B-phase exceed the mean + 2SD in the A-phase indicating a statistically significant improvement, ¤ = at least 2 consecutive assessments in the B-phase exceed the mean - 2SD in the A-phase indicating a statistically significant worsening, # = responder improving ≥ 20% compared to the mean of the A-phase.

**Figure 2:**
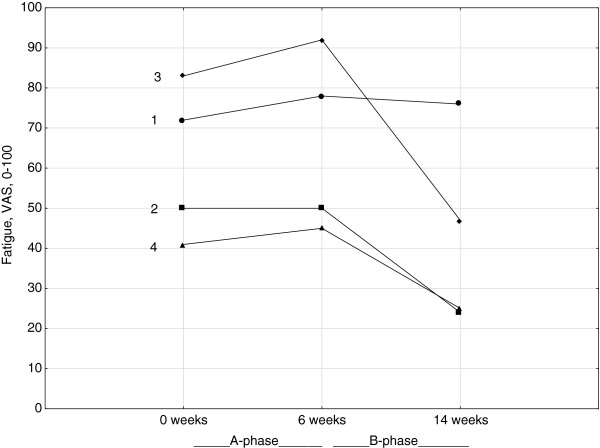
Individual results of patient-reported fatigue assessed by the VAS, 0–100.

By visual graph analysis for trend and slope, a trend towards reduced fatigue was identified in participants 2, 3, and 4 assessed by the VAS which was also supported by an almost statistically significant reduction in fatigue (p = 0.056) (Figure 2). There was no visually identified trend or slope regarding Raynaud’s phenomenon or global disease impact on well-being assessed by the VAS, or activity limitations assessed by the HAQ.

## Discussion

Four participants with SSc and with 54–100% of maximal vital capacity participated in an eight week intensive aerobic and muscle endurance exercise program. Three participants improved their muscle endurance and two participants improved their estimated oxygen uptake. There were no changes in physical capacity, limitations in daily activities, quality of life, Raynaud’s phenomenon or global disease impact on well-being, although there was a trend toward reduced fatigue following the exercise program.

There was no change in the primary outcome physical capacity measure 6MWT in any of the patients as there were large variations in individual walking distances during both the A- and the B-phase. Studies have pointed out that lower-extremity joint or muscle-oriented pain, as well as central and peripheral exertion also could reduce the walking distance [29,30]. This was the case with participants 3 and 4 in our study, who had fluctuating arthritis of the ankles and reduced balance and tactility of the distal lower limbs due to spinal stenosis, respectively. Participant 2 was unable to complete the treadmill test during the A-phase assessments, making it impossible to calculate an A-phase mean and 2 SD. However, as this participant was able to complete all tests during the B-phase, this was interpreted as a clinically relevant improvement. Since most participants performed the aerobic exercise on a stationary bike, it would probably have been better to perform the estimated oxygen uptake test on a bike. However, prior clinical experience suggested that lower limb muscle fatigue might hamper patients’ ability to complete a submaximal oxygen uptake biking test. Further research is needed to find a valid and feasible method to measure aerobic capacity and physical capacity in SSc and when using the 6MWT in clinical practice and research in these patients, a careful consideration of its measurement properties is important.

There was also a within-patient variation in muscle endurance in different tasks of the FI-2. Participants 2 and 3 improved significantly in either shoulder flexion or hip flexion. Participant 4 had a large variation in numbers of repetitions in the right hip flexion task, producing a wide 2SD-band. Although the participant performed clinically relevant more repetitions during assessments in the B-phase compared to the mean value of assessments in the A-phase, the wide 2SD-band prevented this change from reaching statistical significance. Although reaching the maximal number of repetitions of shoulder flexion during the A-phase, this participant was able to increase the training load from 1 to 2 kg suggesting increased muscle endurance.

The visual plot analysis revealed a trend toward reduced fatigue in three participants. Fatigue is one of the most disabling symptoms in SSc [6]. Our results suggest that exercise might be an effective treatment to reduce fatigue and this should be a prioritized research area in the future.

Four months prior to entering the study, participant 1 had stopped the treatment with mycophenolate mofetil, which is the most probable explanation for the increased lung symptoms present in the beginning of the B-phase. As the participant had exercised with a similarly intensive program about a year before entering the study, and while participating in this study never reached the intensive exercise equivalent with perceived exertion of 15 on the Borg scale, it is not likely that the exercise program contributed to increased lung symptoms. This is supported by studies reporting the safety of other inflammatory systemic conditions such as myositis and SLE [15,31] and prior studies reporting the safety of moderate exercise intensity in SSc with lung fibrosis [12,13]. Participant 1, with the most severe lung involvement, did not improve, while the participant 2, with slightly milder lung fibrosis, did improve in both aerobic capacity and muscle endurance. This might emphasize the importance of optimal medical treatment to achieve the positive effects of exercise in patients with SSc and lung fibrosis. Due to differences in study populations, numbers of participants, exercise programs and climates it is difficult to compare our results with prior studies evaluating exercise in SSc. Our study was conducted during the winter or early spring when patients with SSc usually experience more Raynaud’s phenomenon [32]. Since no participant in our study experienced increased Raynaud’s phenomenon, this might in fact be an effect of the exercise. Our results confirm previous reports of intensive exercise in SSc without lung fibrosis leading to improved or unchanged aerobic capacity, improved muscle function or the absence of improvements in quality of life assessments and adverse events (10, 11). However, our study is the first to reveal data indicating that exercise might reduce fatigue in SSc.

All patients were familiar with the objective and the self-reported measures included in this study as they are all used in standard clinical follow-ups as well as in the ongoing screening study at the Rheumatology clinic, Karolinska.

A SSED was used for this study as we predicted difficulties in recruiting enough patients for a randomized controlled trial (RCT). As a statistical analysis can be performed for each individual, a SSED could have higher validity compared to an open-label group analysis since similar improvements were achieved among three of the participants [27,28]. Using the ABA-design, also including a new non–interventional A-phase following the B-phase, would have further strengthened our study. Following recommendations from the regional ethical committee, the AB-design was used as it might be unethical to ask patients to discontinue a potentially effective treatment. Physical tests were performed every other week to avoid training effects during the A-phase. The variation in physical capacity was probably due to day-to-day variations and the changes in temperature and humidity. Our experience with using a SSED in an exercise pilot study involving patients with polymyositis and dermatomyositis revealed less day-to-day variations producing smaller 2 SD-bands [33]; this type of design has also been used successfully in an exercise study in SLE [31].

Exercise intensity was set according to participants’ subjective ratings of exertion using the Borg RPE scale, which is a limitation as there might be individual differences in the perception of perceived exertion [21].

Only four participants were included, limiting the external validity of the study with the majority who chose not to participate being younger and still working. As fatigue is a common symptom (6), maybe they did not have the energy to include a 3-days-a-week exercise program in their daily lives. Perhaps a home-based exercise program would be easier to incorporate into daily life. As all the participants were registered at a university hospital, they might have had a more severe disease course than the general population with SSc. However, both types of diagnosis were represented, and the three women and one man included could be representative as the majority of individuals with SSc are women [4]. To achieve solid evaluation of the safety and efficacy of exercise in SSc, larger multi-center RCTs are needed. There is an ongoing effort to establish a core set of measures to be used in SSc which would enhance these kinds of studies [34].

## Conclusions

In conclusion, this eight week aerobic and muscle endurance exercise program was overall beneficial, resulting in improved aerobic capacity and muscle endurance in two out of four participants. There were no changes in the primary outcome 6MWT or in the patient reported outcomes, although there was a trend towards reduced fatigue. There is a need for a large multi-center RCT, also including patients with various degree of lung involvement to further establish evidence of exercise effects in SSc.

## Abbreviations

lcSSc: Limited cutaneous systemic sclerosis; dcSSc: Diffuse cutaneous systemic sclerosis; ILD: Interstitial lung disease; PAH: Pulmonal hypertension; FVC: Forced vital capacity; 6MWT: Six-minute walking test; FI-2: Functional Index 2; HAQ: Health Assessment Questionnaire; SF-36: Short Form 36; VAS: Visual Analogue Scale; SSED: Single subject experimental design; 2SD band: 2 standard deviation band.

## Competing interests

None of the authors have any competing interests.

## Authors’ contributions

HA, LB and AN conceived the study, HA supervised all exercise sessions and analyzed and interpreted data, JB performed all clinical assessments and analyzed and interpreted data. All authors contributed to the manuscript including reading and approving the final version of the manuscript.
